# Highly Sensitive Measurement of Liquid Density in Air Using Suspended Microcapillary Resonators

**DOI:** 10.3390/s150407650

**Published:** 2015-03-30

**Authors:** Oscar Malvar, Daniel Ramos, Carmen Martínez, Priscila Kosaka, Javier Tamayo, Montserrat Calleja

**Affiliations:** Institute of Microelectronics of Madrid (IMM-CSIC), Isaac Newton 8 (PTM), Tres Cantos, 28760 Madrid, Spain; E-Mails: oscar.malvar@csic.es (O.M.); daniel.ramos@csic.es (D.R.); carmen.martinez.d@csic.es (C.M.); priscila@imm.cnm.csic.es (P.K.); mcalleja@imm.cnm.csic.es (M.C.)

**Keywords:** micromechanical sensors, microcapillaries, suspended microchannel resonators, rheology

## Abstract

We report the use of commercially available glass microcapillaries as micromechanical resonators for real-time monitoring of the mass density of a liquid that flows through the capillary. The vibration of a suspended region of the microcapillary is optically detected by measuring the forward scattering of a laser beam. The resonance frequency of the liquid filled microcapillary is measured for liquid binary mixtures of ethanol in water, glycerol in water and Triton in ethanol. The method achieves a detection limit in an air environment of 50 µg/mL that is only five times higher than that obtained with state-of-the-art suspended microchannel resonators encapsulated in vacuum. The method opens the door to novel advances for miniaturized total analysis systems based on microcapillaries with the add-on of mechanical transduction for sensing the rheological properties of the analyzed fluids without the need for vacuum encapsulation of the resonators.

## 1. Introduction

Measurement of the density and viscosity of liquids is of high importance in the pharmaceutical, chemical, petroleum and food industries. Commercial instruments for measuring the density of liquids are based on vibrating macrosized U-tubes, and the principle is that the resonance frequency decrease is inversely proportional to the mass of liquid that flows through the tube [1]. Sample volumes of milliliters and resonance frequencies of 40–400 Hz are typical in these devices. The limit of detection is ~10 µg/mL. There is an increasing need to perform these analyses with smaller volumes, well below the µL scale, in a wide variety of applications such as on-column analysis in micro high performance liquid chromatography (µHPLC) [2] and capillary electrophoresis (CE) [3], blood plasma analysis [4,5,6] and for monitoring biological and chemical reactions [7,8,9]. Two techniques have emerged for measuring the density and viscosity of liquids based on microsized mechanical resonators [10]: plate microresonators such as microcantilevers (MCs) vibrating inside the liquid sample [5,6,7,8,9,11,12,13] and suspended microchannel resonators (SMRs) [14,15,16]. The first technique is based on the effect of the hydrodynamics interactions between the liquid sample and the MC on the resonant frequency and quality factor of the microcantilever [17,18]. It achieves a limit of detection of about 5 µg/mL, with sample volumes of ~10 µL [12]. The second technique is based on a miniaturization of commercial density sensors, although SMRs are usually shaped as cantilevers instead of being U-shaped. Since the liquid is inside the device, the SMRs can be measured in vacuum in order to minimize the viscous damping, and thereby to boost the frequency resolution [19]. SMRs represent the state-of-the art in density sensors as they can analyze liquid volumes of 5–10 pL with detection limits of 4–10 µg/mL [14,15]. However, SMRs remain non-affordable and unavailable to most laboratories due to the microfabrication complexity, microfluidics integration and the vacuum encapsulation.

In this work, we present a density sensor based on commercially available microcapillaries resonating in air and an optical detection approach based on the measurement of the forward scattering of a laser beam shined on the microcapillary. The technique allows the measurement of the density of nanoliter amounts of sample with sensitivity comparable to state-of-the-art technologies but without the trouble of the vacuum encapsulation of the devices. Moreover, these devices can be easily integrated with µHPLC and CE for on-column analysis and with flow cytometers for studying the rheology of biological fluids.

## 2. Results and Discussion

**Figure 1:**
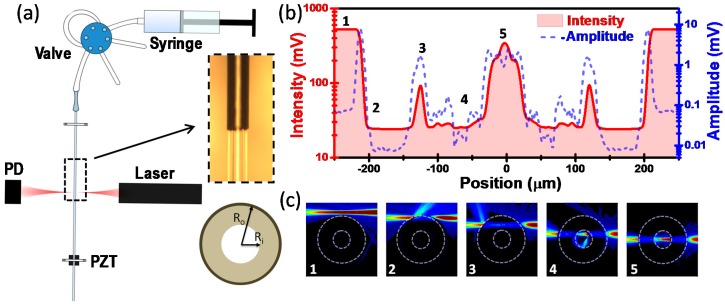
(**a**) Schematic depiction of the experimental setup. The reference liquid is injected into the microcapillary by means of a syringe pump. The liquid samples are introduced in the fluidic circuit by a mixing valve. The optical setup consist of a laser diode facing a photodiode, which collects the light scattered by the microcapillary. The inset shows an optical micrograph of a region of the capillary with and without the polyimide coating at the top and bottom regions, respectively; (**b**) Calibration of the optical transduction of the microcapillary displacement. The graph shows the DC component and AC component at the capillary resonance frequency of the photodiode output voltage as a function of the transversal distance between the microcapillary and the laser beam; (**c**) Finite element method simulations of the electromagnetic field distribution resulting of the interaction of the laser beam and the microcapillary for different positions of the laser beam labelled as 1, 2, 3, 4 and 5 that are identified in (b).

A schematic of the experimental set-up is shown in Figure 1a. Our mechanical resonator is a suspended region of a commercially available fused silica microcapillary that is mechanically clamped at two positions. The distance between the two clamped ends can be accurately controlled by means of micropositioning stages. In this work, we choose a length of 4.5 mm that gives a natural resonance frequency of ≈78 kHz. The inner ($R_{i}$) and outer radii ($R_{o}$) of the microcapillaries are 75 µm, and 187 µm, respectively. The silica capillaries include a 22 µm thick polyimide capping layer that provides flexibility to the capillary, and thereby it eases its handling. The inset in Figure 1a shows a region of the microcapillary, in which the capping layer has been removed. The liquid flow in the microcapillary is controlled by a syringe pump (neMESYS, Cetoni GmbH, Korbussen, Germany) equipped with a low pressure injector valve and an injection loop for introducing the sample solutions. The flow rate was kept at 0.5 µL/s in the present experiments. It is noteworthy that, despite the large size of the capillary in comparison with microcantilevers, the analyzed sample volume, ≈20 nL, is significantly smaller than that with microcantilevers, ~10 µL. The microcapillary displacement is optically detected by focusing a laser beam (3 mW, 639 nm, Schäfer-Kirchhoff GmbH, Hamburg, Germany) on the middle of the suspended region of the capillary (beam waist ≈5–10 µm), collecting the forward scattered light by means of a 10 × 0.28 NA objective (Mitutoyo, Chicago, IL, USA), and measuring the intensity of the collected light by a photodetector (PDA36A-EC, Thorlabs Inc., Newton, NJ, USA). The microcapillary is mechanically driven by a piezoelectric actuator located beneath one of the clamped ends. The microcapillary is driven at the fundamental resonance frequency by using a phase-locked loop configuration (HF2LI-PLL, Zurich Instruments, Zurich, Switzerland).

The principle of the displacement detection technique is that the amount of light scattered by the microcapillary depends sensitively on its position, and thereby, the displacement of the microcapillary results into a variation of the optical power collected by the photodetector. The displacement was calibrated by measuring the photodetector output voltage as a function of the transversal distance between the laser beam and the microcapillary (Figure 1b). In the experiment, the microcapillary was driven at its resonance frequency, and the resulting photodetector voltage amplitude was also detected. As expected, there is a correlation between the amplitude and the absolute value of the voltage slope. For the sake of major understanding, we calculated the distribution of the optical intensity by the finite element method (Comsol Multiphysics, Palo Alto, CA, USA) for several relative positions of the laser beam with respect to the microcapillary (Figure 1c). We identify two optimal regions for measuring the microcapillary vibration: near the edge and close to the center of the capillary (labeled as 2 and 5 in Figure 1b,c, respectively). The experiments presented here were carried out with the laser beam focused on near the edge of the microcapillary, where the photodetector voltage amplitude exhibits a maximum. In this configuration, the optical responsivity defined as the relative change of collected optical power with respect to the position of the microcapillary was of 0.132 $\text{μ}m^{-1}$, and the displacement noise ≈10 nm $/\sqrt{Hz}$.

**Figure 2:**
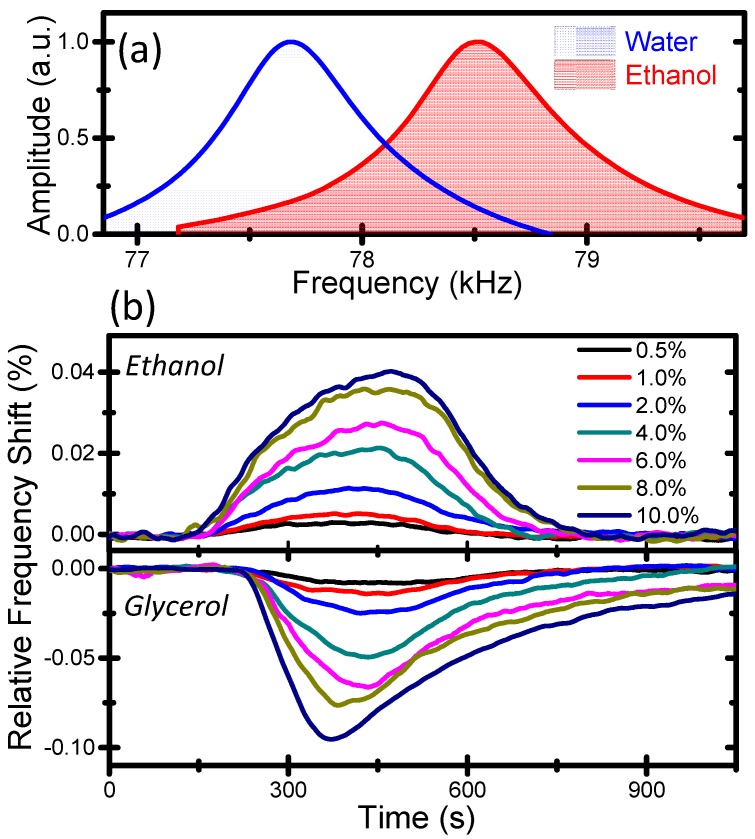
(**a**) Resonance frequency peak of the microcapillary filled with water and ethanol; (**b**) Series of real-time measurements of the relative change of the resonance frequency for binary mixtures of ethanol and glycerol in water for volume concentrations from 0.5% to 10%. The binary mixtures were injected in water that is the reference liquid in these experiments.

Figure 2a shows the fundamental resonance frequency peak of the microcapillary filled with water (ρ ≈ 998 Kg/m^3^) and ethanol (ρ ≈ 789 Kg/m^3^). The vibration amplitudes, ≈400 nm, are well below the capillary diameter, and therefore nonlinear effects on the resonance frequency are negligible. The resonance frequency for the microcapillary filled with water is 77.700 kHz, and it increases to 78.540 kHz when filled with ethanol as a consequence of the lower density of the ethanol. The quality factor is of about 130, and it is dominated by the hydrodynamic interaction between the capillary and air [17]. Figure 2b shows a series of real-time measurements of the relative change of the resonance frequency for binary mixtures of ethanol and glycerol in water for volume concentrations from 0.5% to 10%. Notice that the total volume of the mixture remains smaller than the sum of their individual volumes due to polar nature of ethanol and water molecules [20].The binary mixtures were injected in water that is the reference liquid in these experiments. As soon as the mixture reaches the suspended region of the microcapillary, the resonance frequency starts to change up to achieving a maximum that corresponds to the highest concentration of the mixture in the capillary, then the resonance frequency variation decreases up to achieving the baseline value corresponding with the capillary filled with water. The process lasts about 10 min that approximately corresponds with the mixture volume (300 µL) divided by the flow rate (0.5 µL/s). The lack of a plateau in the resonance frequency measurement is related to the diffusion of the injected mixture with water in the fluidic circuit, which could be avoided by using an immiscible oil drop in between the reference and the mixture sample. As expected, the investigated binary mixtures give opposite shifts in the resonance frequency, ethanol is less dense than water whereas glycerol is denser (ρ ≈ 1261 Kg/m^3^).

**Figure 3:**
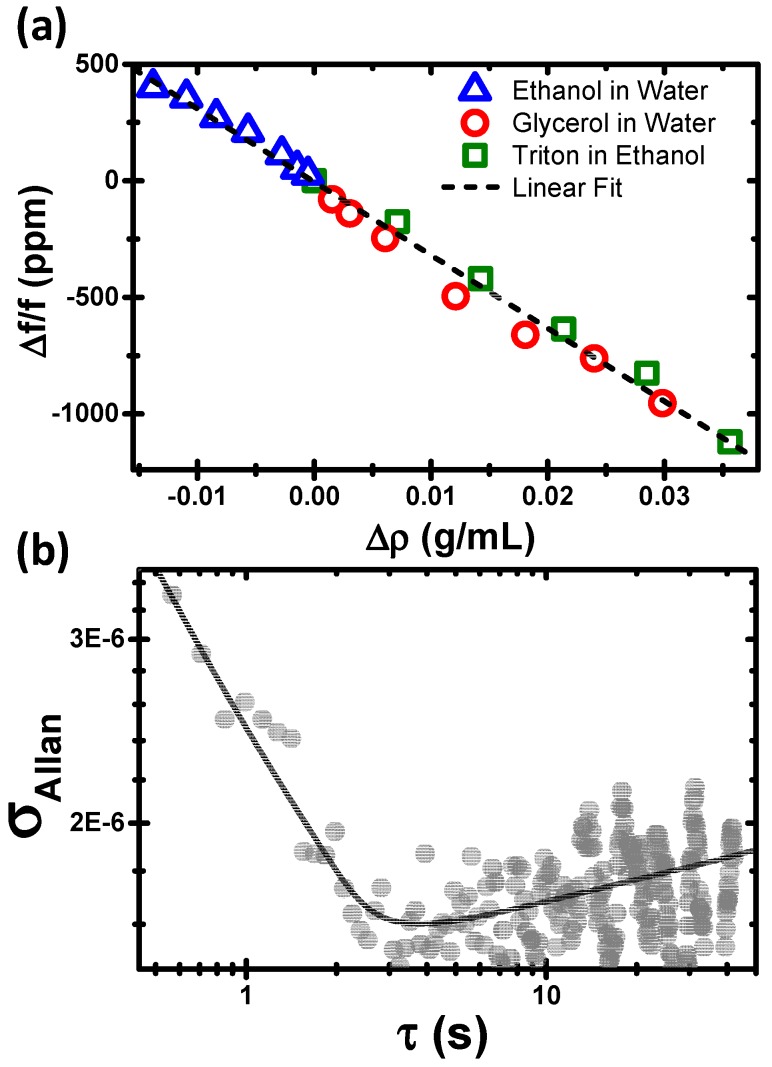
(**a**) Maximal variation of the relative resonance frequency shift *versus* the density variation (symbols). The data is obtained for three binary mixtures: ethanol in water, glycerol in water and Triton-X100 in ethanol; at different concentrations ranging from 0.5% to 10%. The data for the three mixtures is fitted to a straight line whose slope provides the responsivity of the device; (**b**) Allan variance of the resonance frequency of the device *versus* the averaging time.

The responsivity of the device is characterized by plotting the maximal shift of the relative resonance frequency *versus* the density variation (Figure 3a). The scatter data is obtained from three binary mixtures: ethanol in water, glycerol in water and Triton-X100 (ρ ≈ 1070 Kg/m^3^) in ethanol; at different concentrations ranging from 0.5% to 10%. The data for the three mixtures approximately collapse into a straight line whose slope provides the responsivity of the device (R) that is defined as the absolute value of the ratio between the relative resonance frequency shift and the density variation in g/mL. The experimental responsivity is 0.0314 mL/g. We compare this value with the theoretical responsivity given by:

(1) $$R\equiv \left|\frac{\partial \left(\frac{\Delta f}{f}\right)}{\partial \Delta {\text{ρ}}_{l}}\right|≅-\frac{1}{2}\frac{1}{{\text{ρ}}_{g}\left\{{\left(\frac{R_{0}}{R_{i}}\right)}^{2}-1\right\}+\frac{2{\text{ρ}}_{p}h_{p}R_{0}}{{R_{i}}^{2}}+{\text{ρ}}_{l}}$$

where $R_{0}$ and $R_{i}$ are the outer and inner capillary radii, respectively, $h_{p}$ is the thickness of the polyimide coating layer, ${\text{ρ}}_{g}$, ${\text{ρ}}_{p}$ and ${\text{ρ}}_{l}$ are the density of the capillary, polyimide layer and the liquid inside, respectively. The theoretical responsivity for the microcapillary used in this work is 0.0334 mL/g, *i.e*., 6% higher than the experimentally determined value. This difference is attributed to the diffusion of the injected mixture with the reference liquid that fills the fluidic circuit.

In order to determine the limit of detection in density variation, we characterize the resonance frequency stability by the Allan variance ${\text{σ}}_{A}\left(\tau \right)$, defined as one half of the average of the squares of the differences between successive readings of the frequency deviation separated by a time interval τ (Figure 3b). For time intervals τ<3 s the Allan variance behaves as $\sim \tau^{-0.5}$ indicating that the frequency noise is dominated by white noise processes. For longer time intervals, the Allan variance starts to increase, which indicates that the resonance frequency displays drift. A frequency stability of $1.6\times 10^{-6}$ is achieved for averaging times of 3 s, which divided by responsivity of the device (R) gives a density detection limit of ≈50 μg/mL. Although the limit of detection is between 5 and 10 times higher than that obtained by state-of-the-art suspended microchannel resonators encapsulated in vacuum [14,15,19] or photothermally self-excited microcantilevers in liquid [12], this limitation is well-justified by the low cost and simplicity of the experimental set-up. Moreover, the technique has room for larger improvement by enhancing the frequency stability and density responsivity. Thus, it is expected that the frequency stability can be enhanced one order of magnitude by operating the device in moderated vacuum. On the other hand, it is clear that by examining Equation (1) the responsivity can be largely enhanced by decreasing the relative thickness of the microcapillary wall, (*R*_0_‒*R_i_*)/*R_i_* that in our current experimental set-up is ≈1.5, very far from the optimal value. A responsivity enhancement of 5 times is achieved by thinning the glass walls to 15 μm (*R*_0_‒*R_i_*)/*R_i_* ≈ 0.2.

## 3. Conclusions

In conclusion, we demonstrate that suspended microcapillary resonators can be used as sensitive density sensors. The capillary vibration is detected by measuring the optical forward scattering, which can be carried out with an accessible set-up based on a laser diode and a photodiode. Microcapillaries are omnipresent in a wide variety of applications in life sciences and chemical analysis and therefore they are commercially available with a wide range of geometries and sizes. The results of this work open the door to novel advances for miniaturized total analysis systems based on microcapillaries with the add-on of mechanical transduction for sensing the rheological properties of the analyzed fluids.
